# Acute Effects of Aerobic Exercise on Executive Function and Attention in Adult Patients With ADHD

**DOI:** 10.3389/fpsyt.2019.00132

**Published:** 2019-03-26

**Authors:** Aylin Mehren, Jale Özyurt, Alexandra P. Lam, Mirko Brandes, Helge H. O. Müller, Christiane M. Thiel, Alexandra Philipsen

**Affiliations:** ^1^School of Medicine and Health Sciences, Psychiatry and Psychotherapy, University Hospital Karl-Jaspers-Klinik, University of Oldenburg, Oldenburg, Germany; ^2^Biological Psychology Lab, Department of Psychology, European Medical School, University of Oldenburg, Oldenburg, Germany; ^3^Department of Psychiatry and Psychotherapy, University of Bonn, Bonn, Germany; ^4^Unit Applied Health Intervention Research, Department of Prevention and Evaluation, Leibniz Institute for Prevention Research and Epidemiology - BIPS GmbH, Bremen, Germany; ^5^Research Center Neurosensory Science, University of Oldenburg, Oldenburg, Germany; ^6^Cluster of Excellence “Hearing4all,” University of Oldenburg, Oldenburg, Germany

**Keywords:** acute aerobic exercise, adult ADHD, brain activation, cardiorespiratory fitness, cognition, executive function, fMRI, physical activity

## Abstract

Aerobic exercise can improve cognitive functions in healthy individuals and in various clinical groups, which might be particularly relevant for patients with ADHD. This study investigated the effects of a single bout of aerobic exercise on attention and executive functions in adult patients with ADHD, including functional MRI to examine the underlying neural mechanisms. On two different days, 23 adult patients with ADHD and 23 matched healthy controls performed in a flanker task, while functional MR images were collected, following 30 min of continuous stationary cycling with moderate intensity as well as after a control condition (watching a movie). Behavioral performance and brain activation were tested for differences between groups and conditions and for interactions to investigate whether exercise improves executive function to a greater extent in patients compared to healthy controls. Exercise significantly improved reaction times in congruent and incongruent trials of the flanker task in patients with ADHD but not in healthy controls. We found no changes in brain activation between the two conditions for either group. However, a subgroup analysis of ADHD patients with a higher degree of cardiorespiratory fitness revealed decreased activation in premotor areas during congruent trials and in premotor and medial frontal cortex during incongruent trials in the exercise compared to the control condition. Our results indicate exercise-induced improvements in attention and processing speed in patients with ADHD, demonstrating that adult patients with ADHD may benefit from an acute bout of exercise. These findings could be of high relevance for developing alternative treatment approaches for ADHD. In addition, results of the current study contribute to elucidate the neurophysiological mechanisms underlying the beneficial effects of exercise on cognition and to better understand the role of cardiorespiratory fitness on these effects.

## Introduction

Attention deficit hyperactivity disorder (ADHD) is well-known as a disorder affecting children, even though symptoms often persist until adulthood (1). Impairments in executive function, e.g., difficulties to sustain attention, increased distractibility, or reduced control of response interference are common among patients with ADHD (2), affecting various areas of daily life. Adult patients often report difficulties at work or in social communication, situations in which attentional and interference control are crucial (3). Experimental paradigms that have been developed to measure interference control include the Stroop task, Eriksen flanker task, and the Simon task. There are numerous studies demonstrating that patients with ADHD show impaired performance in these tasks compared to healthy participants (4–7). Neuroimaging studies have further reported abnormal brain activation in patients during executive function tasks, mainly affecting fronto-striatal and parietal regions, but also temporal and subcortical structures (8–12). However, there is no consensus on the direction of activation differences (11–14).

Aerobic exercise might be effective in improving executive function in ADHD. Studies in healthy participants as well as patients with several pathological conditions including ADHD have shown that both chronic and acute exercise can improve executive functioning, attention, as well as symptoms of hyperactivity and impulsivity (15–19). The majority of studies concentrating on children with ADHD have found positive effects of acute exercise on different aspects of executive function [e.g., (20–23); see (24) for review], whereas there are only two studies on adult patients revealing mixed results. Gapin et al. (25) tested college students with and without ADHD before and after an acute bout of exercise and found improved inhibitory control during the Stroop task after exercise in both groups. This study, however, suffers from methodological caveats, including only 10 participants per group. Furthermore, testing before and after an exercise session might lead to behavioral improvements reflecting habituation or practice effects. In contrast, Fritz and O'Connor (26) included a resting control condition and reported exercise-induced improvements in mood and motivation in 32 adult male patients, but no changes in attention and hyperactivity.

Proposed neurophysiological mechanisms underlying the cognitive benefits of acute exercise include increases in arousal and catecholamine levels (27–29). These mechanisms are mainly associated with prefrontal cortex functioning which might explain why executive functions seem to benefit more from exercise than other cognitive functions (16, 30–33). Furthermore, increases in cerebral blood flow (CBF) due to exercise have been observed (34, 35), while it is not clear yet if these changes are specific to prefrontal brain regions or rather global. Some authors speculate that exercise could elevate the proportion of CBF in prefrontal brain regions in comparison to other areas (36). Note that CBF changes have mainly been assessed during exercise [e.g., (34)] and results from studies measuring CBF following an acute bout of exercise are scarce and inconsistent (35–38).

Very few studies investigated brain activation during cognitive tasks following acute exercise in healthy individuals using fMRI. Recently, two fMRI studies reported exercise-induced increases in activation of parietal and hippocampal regions (39) and increases as well as decreases in activation in frontal regions (40) during working memory performance as assessed by an N-back task. MacIntosh et al. (37) found decreased activation in the left parietal operculum during a Go/No-go task post exercise compared to pre exercise. In psychiatric patients, only one neuroimaging study investigated the acute effects of exercise on cognition, revealing exercise-induced deactivation in frontal and subcortical brain regions during a sustained attention task in patients with bipolar disorder, which was stronger than in healthy controls. However, no behavioral effects were found (41). To our knowledge, no fMRI studies of acute exercise effects in patients with ADHD have been conducted. However, Choi et al. (42) included fMRI to test the effects of a 6 week exercise program in addition to methylphenidate treatment on Wisconsin Card Sorting test performance in adolescents with ADHD and found that exercise in addition to medication increased frontal lobe activity to a greater extent than medication only.

Previous studies showed that exercise influences different areas of cognitive processing in a differential manner, with several moderating factors playing a significant role. Examples for such factors are the timing of cognitive task administration, which might interfere with the duration of exercise effects, and the fitness level of the participants, which might interact with neurophysiological responses and contribute to behavioral effects (15, 16, 36, 43). Higher levels of fitness have been associated with enhancements in brain structure, function, and cognitive performance [for reviews, see (44–47)]. In addition, a few studies investigated the association between fitness and acute exercise effects on cognition. Hogan et al. (48) reported cognitive benefits of acute exercise for healthy adolescents with higher fitness levels as compared to those with lower fitness levels. Tsai et al. (49) included neuroelectric measures to test the effects of acute exercise on the performance in a visuospatial attention task in healthy young adults. While exercise improved behavioral performance and increased central Contingent Negative Variation (CNV) area irrespective of fitness level, only the higher fit group showed increases in P3 amplitude and frontal CNV area, which are indicators of allocation of attention and cognitive preparation, respectively. These findings were supported by another study by Tsai et al. (50), demonstrating that only higher fit participants showed exercise-related increases in P3 amplitude during a task-switching paradigm. Nevertheless, previous studies investigating acute exercise effects on patients with ADHD or on brain activation as measured by fMRI have not considered fitness as a moderating factor.

The aim of the current fMRI study was to investigate acute effects of aerobic exercise on executive functioning and associated brain activation in adult patients with ADHD. Therefore, patients and healthy controls performed in a flanker task in the MRI scanner in two conditions: Following an exercise condition requiring 30 min of continuous cycling at moderate intensity, and following a control condition requiring to watch a movie. We hypothesized that exercise improves executive function as reflected in better task performance and altered brain activation in the exercise compared to the control condition in patients and in healthy controls. Based on previous studies showing that individuals with lower cognitive performance levels are more susceptible to benefits from exercise (15, 30, 51), we expected that patients would benefit to a greater extent. On an exploratory basis, we further assumed that individual fitness levels of participants are associated with the amount of cognitive facilitation induced by the exercise intervention.

## Materials and Methods

### Participants

Data acquisition was conducted in the context of a larger project on acute exercise effects on performance in different cognitive tasks in patients with ADHD and healthy controls. For the purpose of the current study, we recruited a total of 46 adult participants (23 patients with ADHD and 23 healthy controls). ADHD patients were recruited through the outpatient clinic of the Department of Psychiatry and Psychotherapy at the University of Oldenburg. Healthy control participants were recruited from announcements in the internet. All patients had received the diagnosis of ADHD according to international guidelines (NICE guidelines; nice.org.uk/guidance/cg72), based on the diagnostic criteria of the Diagnostic and Statistical Manual of Mental Disorders (4th ed.; DSM-IV; (52)). Patients were diagnosed by a trained psychiatrist following a detailed psychiatric interview that integrates somatic differential diagnosis and the patients' medical history. Following tests were used to confirm the diagnosis: the German versions of the ADHD Self Rating Scale (ADHS-SB) (53), Wender Utah Rating Scale (WURS-k) (54), Conners' Adult ADHD Rating Scale–Self-Report: Long Form (CAARS-S:L) (55), and Symptom-Checklist-90 (SCL-90-R) (56). Healthy controls were age- and gender-matched to the patients. ADHD symptoms were assessed by the ADHS-SB and the WURS-k.

Exclusion criteria for all participants were (i) neurological or severe psychiatric disorders, as assessed with the German versions of the Structured Clinical Interview for DSM-IV (SCID-I) (57), the SCID-II screening questionnaire for personality disorders (58), and the Beck Depression Inventory (BDI-II) (59), (ii) autism spectrum disorders, and (iii) psychotropic drugs (for patients: psychotropic drugs different from medication for ADHD). Further exclusion criteria were MRI contraindications and health conditions that interfere with exercise safety. To evaluate health conditions, an electrocardiogram and a health questionnaire (German version of the Physical Activity Readiness Questionnaire) (60) were completed before participating. One subject had to be excluded from the analysis due to misconceptions concerning task performance, albeit he had shown seemingly correct performance during the training task. Two further patients had to be excluded due to excessive head motion during the MRI (i.e., scan-to-scan or total displacement higher than 3 mm), so that 40 participants (20 patients and 20 matched controls) were included in the analyses. The demographic and clinical characteristics of these 40 participants are summarized in Table 1.

**Table 1:** Demographic and clinical characteristics among patients with ADHD and healthy controls.

**Variable**	**ADHD (*n* = 20) mean ± SD**	**Controls (*n* = 20) mean ± SD**	**Statistic**	***P*-value**
Age (years)	29.9 ± 9.5	29.0 ± 7.4	*t* = 0.32	0.75
Gender (f/m)	4/16	5/15	X^2^ = 0.14	0.71
BMI (kg/m^2^)	25.0 ± 3.8	24.3 ± 2.7	*t* = 0.64	0.53
HR_max_ (beats/min)	179.9 ± 9.6	187.2 ± 10.1	*t* = −2.33	0.025^*^
VO_2peak_ (mL/min/kg)	37.1 ± 7.2	41.5 ± 7.3	*t* = −1.94	0.060
VO_2peak_ (% ranking)	40.3 ± 22.8	51.0 ± 21.7	*t* = −1.53	0.14
BDI	9.3 ± 5.4	2.3 ± 2.8	*t* = 4.82	<0.001^*^
ADHS-SB	31.4 ± 8.2	5.0 ± 4.4	*t* = 12.71	<0.001^*^
WURS-k	40.4 ± 15.1	8.4 ± 6.8	*t* = 8.65	<0.001^*^
PA total score	8,775 ± 8,922	4,044 ± 3,478	*t* = 2.21	0.037^*^
PA work	4,941 ± 8,349	235 ± 662	*t* = 2.45	0.025^*^
PA transportation	1,582 ± 2,489	973 ± 863	*t* = 1.03	0.31
PA domestic	856 ± 1,035	1,005 ± 2,425	*t* = −0.25	0.80
PA leisure	1,643 ± 1,776	1,831 ± 1,840	*t* = −0.33	0.75
PA walking	1981 ± 2247	608 ± 743	*t* = 2.59	0.016^*^
PA moderate	3,390 ± 3,378	2,118 ± 2,508	*t* = 1.35	0.18
PA vigorous	3,404 ± 5,989	1,318 ± 1,832	*t* = 1.49	0.15
Stimulant medication^a^	*n* = 4	—	—	—

* p <0.05.

a *current medication, discontinued 48 h prior to each visit. BMI, body mass index; HR_max_, maximal heart rate as assessed by maximal exercise test; VO_2peak_, peak oxygen consumption as tested by maximal exercise test; VO_2peak_ (% ranking), peak oxygen uptake transformed into age- and gender-adapted percentiles; BDI, Beck Depression Inventory; ADHS-SB, ADHD Self Rating Scale; WURS-k, Wender Utah Rating Scale, retrospective assessment of childhood ADHD; PA, Physical activity as assessed by the International Physical Activity Questionnaire, expressed in MET-minutes/week*.

The study was conducted in accordance with the Declaration of Helsinki (61) and all procedures were approved by the ethics committee of the University of Oldenburg. All participants gave their written informed consent prior to study participation.

### Experimental Tasks

The stimulus presentation was programmed with Cogent 2000 v125 (http://www.vislab.ucl.ac.uk/cogent.php) and run in Matlab R2015b (The MathWorks, Inc.). The paradigms were projected onto a screen and presented to the participants in the scanner through a mirror on the head coil. All stimuli were white and were displayed on a black background. Participants responded with their right hand via an MR-compatible keypad (NAtA Technologies, Coquitlam, Canada).

#### Flanker Task

**Figure 1:**
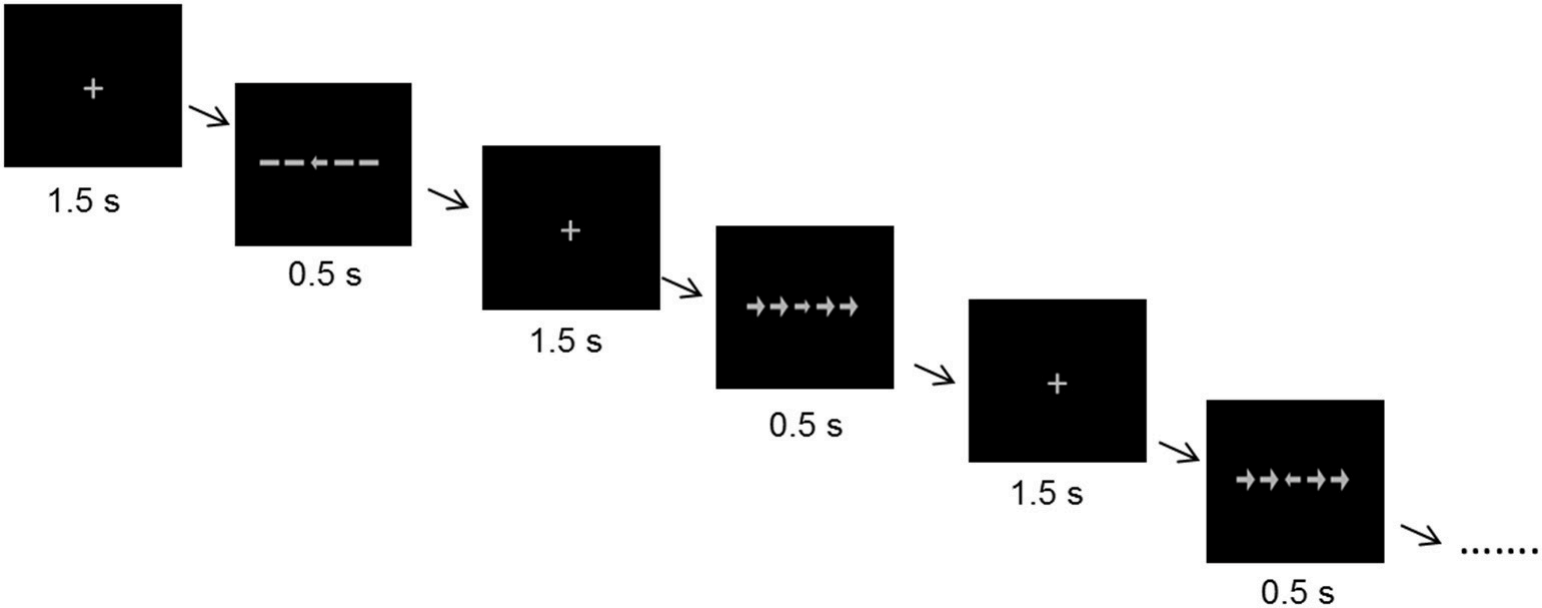
Stimuli and time course of the flanker task.

An arrow version of the Eriksen flanker task (62) was used to measure selective attention and interference control. Each stimulus consisted of 5 arrows in a row (one target at the center and two flankers on either side). The target pointed to the left or to the right with equal probability. We included three different trial types. In congruent trials the flankers were pointing into the same direction as the target, in incongruent trials they were pointing into the opposite direction, and in neutral trials lines without arrowheads served as flankers. To introduce a stronger interference effect, the flankers were larger than the target stimulus. Stimuli were presented in an event-related design. Each trial lasted 2 s, starting with a 1.5 s presentation of a fixation cross which was followed by a stimulus for 0.5 s (Figure 1). The participants' task was to focus on the fixation cross, and, when the stimulus appeared, to indicate the direction of the target by pressing one of two keys (index finger for left, middle finger for right). They were instructed to respond as fast and accurate as possible. The complete task consisted of 300 trials (100 per trial type) and lasted 10 min. The sequence of the trial types as well as the direction of the target was randomized.

#### Visual Task

To test in an explorative analysis whether changes in brain activation are specific to executive task demands or based on rather unspecific and general changes in CBF, we presented a checkerboard task prior to the flanker task (T1). An additional presentation of the checkerboard task at the end of the MRI session (T2) allowed for a further test regarding the duration of exercise effects. Reversing checkerboard images adopted from Sandmann et al. (63) were presented to the participants. The image was a radial black and white checkerboard consisting of 20 rings which were divided into 18 sectors, each with neighboring sectors of opposite color. The proportion of white pixels (luminance ratio) within the pattern was 0.375. The second image was identical but rotated by 180°. The stimuli were presented in a block-design, starting with a 30 s block of pattern-reversing checkerboards which changed at a reversal rate of 3.3 Hz, followed by a 20 s block of focusing a gray fixation cross. This sequence was presented three times so that the complete paradigm lasted 2.5 min.

### Questionnaires

The subjective amount of physical activity during the last 7 days was assessed using the German long form of the International Physical Activity Questionnaire (IPAQ-LF; www.ipaq.ki.se). This self-report questionnaire contains 27 items asking for the time (number of days and minutes/day) spent on walking, moderate, and vigorous physical activity in four domains of daily life: work, transportation, domestic chores and gardening, and leisure-time. Scoring was conducted using the guidelines provided on the IPAQ website. MET-minutes (minutes weighted by energy requirements of specific activity) per week were calculated for each domain and each intensity level of physical activity, as well as an overall physical activity score was computed.

### Experimental Procedure

To minimize the possibility of practice or habituation effects on the experimental tasks (36), we decided for a repeated measures design with a control condition on a different day without pre-intervention measures. Each subject participated in three sessions, which were separated by at least 2 days to avoid aftereffects of exercising on subsequent sessions. During the first visit participants completed a maximal exercise test, during the following two visits they participated in two experimental sessions (exercise and control condition), which were counterbalanced in their sequence. Participants were instructed to refrain from any physical exercise on the test days. Patients were asked to withdraw from stimulant medication at least 48 h prior to each visit.

#### Maximal Exercise Test

To assess participants' cardiorespiratory fitness and their individual maximal heart rate (HR_max_), they completed a maximal exercise test on a bicycle ergometer. The test started with cycling at 90 W. Each 3 min, the resistance was increased by 40 W until the participant was unable to continue pedaling. The heart rate and oxygen consumption were recorded continuously via a chest strap heart rate monitor (Polar RCX5, Polar Electro Oy, Finland) and a spirometer (Oxycon Mobile, CareFusion, Heidelberg, Germany), respectively. At the end of this test, the participant's maximal heart rate and peak oxygen consumption (VO_2peak_ in mL/min/kg) were documented. To determine cardiorespiratory fitness, VO_2peak_ was transformed into age- and gender-adapted percentiles (VO_2peak_ % ranking), based on normative data given by the American College of Sports Medicine (ACSM) (64).

#### Experimental Sessions

At the beginning of the first experimental session, participants performed a practice trial of the flanker task inside the scanner to familiarize with the experimental task and to ensure correct task performance. Afterwards they performed either in the exercise condition or in the control condition. The exercise condition involved 30 min of continuous cycling on an ergometer with moderate intensity. In accordance with the guidelines of the ACSM (64), moderate intensity was reflected by 50–70% of the individual HR_max_. The heart rate was continuously monitored by way of a chest strap heart rate monitor (Polar RCX5; Polar Electro Oy, Finland) and controlled by the experimenter. In the control condition, participants watched the Movie for the Assessment of Social Cognition (MASC-MCk) (65). In contrast to passive watching of a movie or seated reading, which have been used in previous studies as control conditions, this condition has the advantage of being engaged in a task that is entertaining but not cognitively demanding. The movie involves watching short sequences about young people meeting for a dinner and answering questions related to the actors' mental states. It is similar to a soap opera and is very easy to complete. The participants are told that answers are subjective and that there are no incorrect answers. Performance in the movie took approximately 30 min. Note that behavioral performance in the movie did not differ between patients and healthy controls. After each condition, participants followed the same procedures in the MR scanner. They first performed in the visual paradigm (T1) which was followed by the presentation of the flanker task and subsequently a Go/No-go task (for which results are not reported here). At the end of the session, the visual paradigm was presented again (T2) and a structural scan was acquired.

### fMRI Data Acquisition

Imaging was performed on a 3-Tesla MRI Scanner (Siemens MAGNETOM Verio, Siemens AG, Erlangen, Germany) with a twelve-channel head array and a 3-Tesla MRI Scanner (Siemens Magnetom Prisma) with a 64-channel head array. Each patient and his/her respective matched healthy control were tested on the same scanner. Foam pads were used to minimize head movements. Functional images with BOLD-contrast were acquired using multislice T2^*^-weighted gradient echo planar imaging (EPI) (time of repetition (TR) = 1,750 ms, time of echo (TE) = 30 ms, flip angle (FA) = 80°, Field of View (FoV) = 200 × 200 mm^2^, voxel size = 3.0 mm3, matrix size: 64 × 64). Each experimental task was measured during a separate run. For the flanker task 350 EPI volumes and for each visual task 95 EPI volumes were measured, consisting of 31 3 mm-thick axial slices each. Slices were acquired sequentially with a 1 mm gap. Each volume covered the whole brain with the exception of the lowermost part of the cerebellum. After functional scanning a high-resolution T1-weighted structural image using magnetization prepared rapid gradient-echo (MPRAGE) sequence (1 mm3 isotropic voxels, 176 slices, FoV = 250 × 250 mm, TR = 1900 ms, TE = 2.52 ms, FA = 90°) was obtained.

### Data Analysis

#### Behavioral Analysis

Behavioral data were analyzed with SPSS Statistics 22 (IBM, Armonk, NY, USA). Flanker task trials were divided into the three trial types: congruent, incongruent, and neutral. For each trial type mean reaction times and reaction time variabilities were calculated including only correct trials. Additionally, the proportion of errors and proportion of omissions were calculated, reflecting response accuracy. Interference scores were calculated by subtracting mean reaction times between incongruent and congruent trials (RT_incongruent_ - RT_congruent_). Effects of group and condition were analyzed using a 2 (group: ADHD vs. healthy controls) X 2 (condition: Exercise vs. Movie) mixed factorial repeated measures ANOVA. Condition-specific and group differences were tested using paired *t*-tests and two-sample *t*-tests, respectively. To test for associations between behavioral outcomes and participants' fitness level, we calculated Pearson correlations between behavioral outcomes shown to be significantly different between conditions and VO_2peak_ % ranking values. To test for effects of exercise on interference control, we defined the interference score as a primary outcome measure. As the interference score is a summary measure and exercise might affect several components of executive functioning (e.g., attention and inhibition processes) as well as general processing speed, we also defined reaction times in congruent and incongruent trials separately as primary outcome measures. For those three measures, Bonferroni correction was used to account for multiple comparisons (significance level of *p* ≤ 0.017, two-sided). All other analyses were exploratory, not confirmatory, and were interpreted accordingly. Therefore, the significance level was set to *p* ≤ 0.05 (two-sided) and no adjustment for multiple testing was applied.

#### fMRI Analysis

The data were preprocessed and statistically analyzed using SPM12 (Wellcome Trust Centre for Neuroimaging, London, UK) and Matlab R2016a. Preprocessing involved (i) realignment of the functional images to the mean image volume, (ii) coregistration of the functional and structural images, iii) segmentation of the structural image, (iv) spatial normalization to the standard MNI template brain (Montreal Neurological Institute, Quebec, Canada), and (v) spatial smoothing with an 8 mm three-dimensional, full-width-at-half-maximum (FWHM) Gaussian kernel. For each participant, motion parameters were visually inspected and maxima of scan-to-scan and of total displacement were calculated. More than 3 mm of either scan-to-scan or total displacement were determined beforehand as excessive motion. Two patients were excluded from analysis due to this criterion.

At the single-subject level, we performed standard analyses using the general linear model (GLM) in SPM12. For the flanker task, event-related analyses were conducted. To model task-related signal increases, stick functions that were time-locked to the event onsets were used. The first-level GLM design matrix included five regressors of interest: correct congruent, correct incongruent, correct neutral, errors, omissions; and six motion regressors as nuisance regressors. We were primarily interested in the differential contrast incongruent—congruent as well as congruent and incongruent trials separately, representing brain activity related to interference suppression and attention. For the visual task, hemodynamic responses to watching pattern-reversing checkerboards were modeled as box-car functions with a block length of 30 s convoluted with the HRF, time locked to visual stimulation onsets. The first regressor of the GLM design matrix modeled epochs of visual stimulation against an implicit baseline, the six motion parameters were entered as nuisance regressors. To remove non-physiological low-frequency noise, the data were high-pass filtered at 1/128 Hz. Temporal autocorrelation of the fMRI time series was corrected using an AR(2) model. GLM parameters were estimated using the classical SPM approach (Restricted Maximum Likelihood).

On the group level, two-sample *t*-tests with the differential contrasts of Exercise—Movie and Movie—Exercise were used to test for interaction effects between group and condition. To examine between-group differences for each condition separately, we conducted two-sample *t*-tests for Movie and Exercise. Within-group comparisons between the two sessions were tested using paired *t*-tests. As we expected cardiorespiratory fitness to be a moderator of the expected effects, we divided the sample according to their fitness in a *post hoc* analysis and performed the same analyses as mentioned above for the higher fit group. The initial voxel threshold was set to 0.001 uncorrected. Multiple testing was then accounted for on cluster level, based on a corrected *p*FWE of 0.05. As the effects of acute exercise on brain activation have been rarely examined in fMRI studies and results were variable, we conducted whole-brain analyses for both tasks. Stereotaxic coordinates are reported in MNI space and figures are reported using neurological convention.

## Results

### Demographic and Clinical Data

Participant characteristics are depicted in Table 1. As expected, patients and healthy controls did not differ with respect to age, gender, and BMI, but patients scored higher than controls in ADHD symptoms as well as in depression scores. In subjective assessments of physical activity, patients indicated to walk more and to be more active at work than controls, resulting in a higher total score in the International Physical Activity Questionnaire.

### Timing of Task Administration

The average time interval between the end of exercising and the beginning of the experimental tasks was as follows: 5.9 min (*SD* = 1.2, range 4–10) for the visual task at T1, 9.8 min for the flanker task (*SD* = 1.8, range 7–16), and 33.2 min (*SD* = 2.1, range 30–39) for the visual task at T2.

### Effects of Exercise on Brain Activation and Behavior in the Flanker Task

#### Behavioral Results

**Figure 2:**
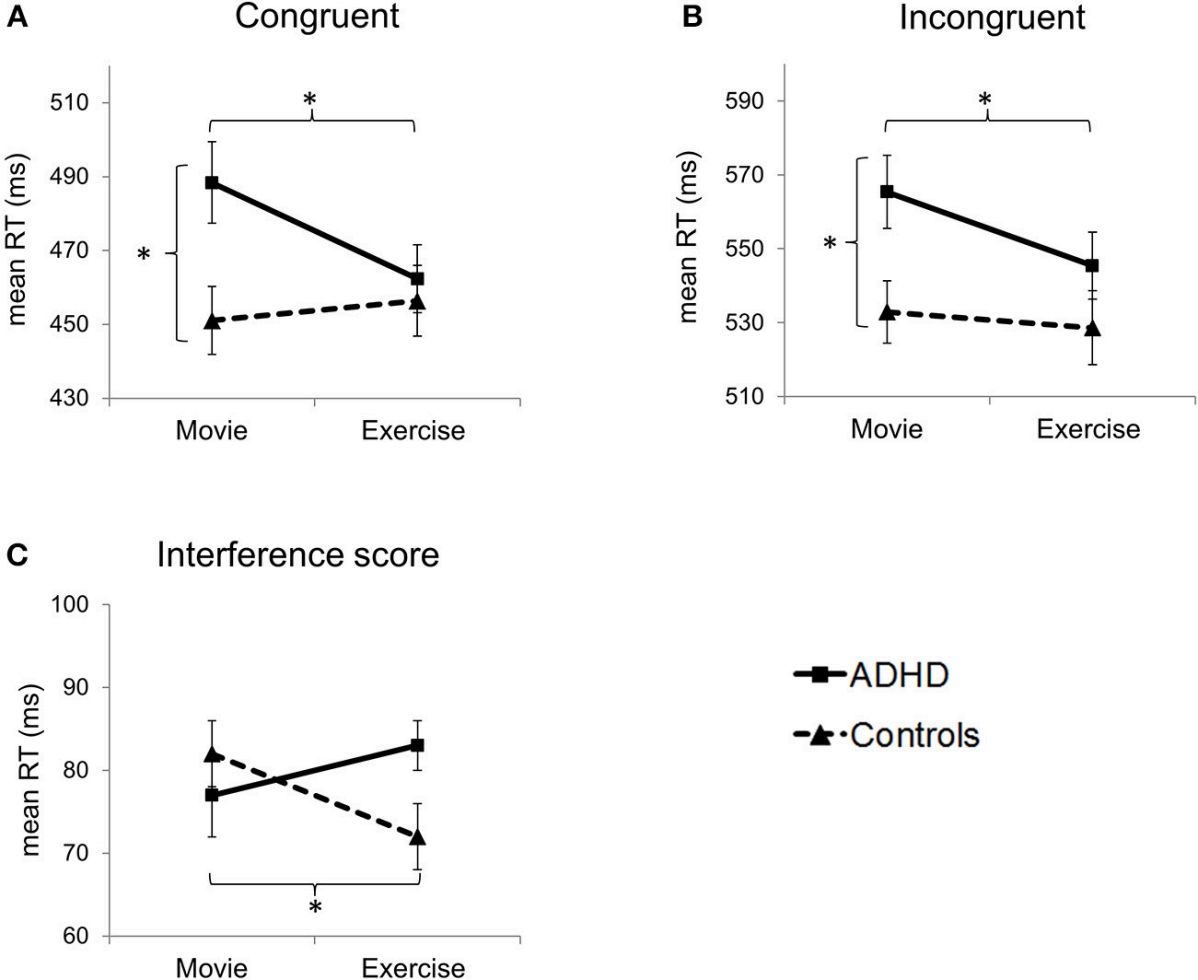
Effects of group and condition on primary outcome measures: reaction times (RT) in **(A)** congruent and **(B)** incongruent trials, and **(C)** interference score. Error bars represent standard errors of the mean. *p < 0.05 (refers to pairwise comparisons, i.e., paired-*t* tests and two-sample *t*-tests).

Group means for all behavioral measures are depicted in Table 2. Main behavioral results are displayed in Figure 2.

**Table 2:** Behavioral performance during the flanker task for each group and condition.

**Variable**	**ADHD (*****n*** **=** **20)**		**Controls (*****n*** **=** **20)**	
	**Movie mean (SE)**	**Exercise mean (SE)**	**Movie mean (SE)**	**Exercise mean (SE)**
RT congruent (ms)	488 (11)	462 (9)	451 (9)	456 (10)
RT incongruent (ms)	565 (10)	545 (9)	533 (8)	529 (10)
RT neutral (ms)	507 (11)	480 (10)	468 (9)	467 (9)
Interference Score (ms)	77 (5)	83 (3)	82 (4)	72 (4)
RTV congruent (ms)	77 (5)	64 (4)	65 (7)	75 (6)
RTV incongruent (ms)	80 (7)	66 (3)	72 (6)	69 (5)
RTV neutral (ms)	74 (6)	59 (3)	65 (6)	69 (5)
Error rate	0.034 (0.009)	0.032 (0.009)	0.032 (0.005)	0.030 (0.005)
Omission rate	0.008 (0.003)	0.002 (0.001)	0.005 (0.002)	0.007 (0.002)

*RT, reaction time; RTV, reaction time variability; SE, standard error of the mean*.

##### Primary outcome measures: reaction times

Interactions between the factors group and condition were found for reaction times in congruent [*F*_(1, 38)_ = 9.28, *p* = 0.004, η^2^ = 0.20] but not in incongruent trials [*F*_(1, 38)_ = 2.63, *p* = 0.11, η^2^ = 0.06]. For congruent trials, the main effect of condition was near significance [*F*_(1, 38)_ = 4.04, *p* = 0.052, η^2^ = 0.10], but there was no main effect of group [*F*_(1, 38)_ = 2.83, *p* = 0.10, η^2^ = 0.07]. For incongruent trials, there was a significant main effect of condition [*F*_(1, 38)_ = 6.30, *p* = 0.016, η^2^ = 0.14] and a near-significant main effect of group [*F*_(1, 38)_ = 3.99, *p* = 0.053, η^2^ = 0.10].

*T*-tests showed significant between-group differences for both trial types in the control condition (Movie), with slower reaction times for patients compared to healthy controls [congruent: *t*_(38)_ = 2.59, *p* = 0.014, *d* = 0.82; incongruent: *t*_(38)_ = 2.50, *p* = 0.017, *d* = 0.79]. Contrary, in the exercise condition, between-group differences in reaction times were no longer significant (*p* > 0.05). These results were due to patients' significantly enhanced reaction time performance in the exercise compared to the control condition [congruent: *t*_(19)_ = 3.64, *p* = 0.002, *d* = 0.81; incongruent: *t*_(19)_ = 2.89, *p* = 0.009, *d* = 0.65], and a similar reaction time performance of healthy controls in both conditions (*p* > 0.05).

Interactions between the factors group and condition were also found for the interference score [incongruent–congruent; *F*_(1, 38)_ = 8.54, *p* = 0.006, η^2^ = 0.18], but there was no main effect of group or condition (*p* > 0.05). Healthy controls showed a decreased interference score after exercise [*t*_(19)_ = 2.73, *p* = 0.013, *d* = 0.61], due to a slightly higher reaction time for congruent trials [*t*_(19)_ = −0.72, *p* = 0.48, *d* = 0.16] and a slightly lower reaction time for incongruent trials [*t*_(19)_ = 0.64, *p* = 0.53, *d* = 0.14].

##### Reaction time variability

Interactions between the factors group and condition were significant for reaction time variability in congruent trials [*F*_(1, 38)_ = 8.29, *p* = 0.007, η^2^ = 0.18]. *T*-tests revealed that patients' reaction time variability in congruent trials decreased in the exercise compared to the control condition [*t*_(19)_ = 3.47, *p* = 0.003, *d* = 0.78]. For incongruent trials there was a near-significant main effect of condition [*F*_(1, 38)_ = 3.56, *p* = 0.067, η^2^ = 0.09] due to numerically decreased reaction time variability after exercise in patients.

##### Accuracy

To explore whether potential changes in reaction times were related to a change in speed-accuracy trade-off, we tested in explorative analyses whether accuracy was affected by exercise. No significant effects were obtained for the error rate or omission rate.

##### Correlation with fitness

We found a significant positive correlation of VO_2peak_ with behavioral outcomes (reaction time differences between Exercise and Movie) in incongruent trials (*r*^2^ = 0.233, *p* = 0.031) in patients, but not in healthy controls.

Note that results for neutral trials are similar to those for congruent trials (see Table 2 for descriptive data).

#### fMRI Results

We were primarily interested in the difference in brain activation between the exercise and control condition. However, to test whether performance in the flanker task revealed brain activation patterns similar to those reported in previous literature, we first plotted brain activation specific to interference control (contrast incongruent–congruent) separate for the exercise and control condition. Main activations were found in frontal and sensorimotor areas, replicating the findings of previous studies (see Supplementary Material). Analyzing the effects of exercise on brain activation in the flanker task, we observed neither interaction effects between group and condition nor within-group effects of condition for any contrast of interest (incongruent–congruent, congruent, incongruent).

### Subgroup Analysis of Participants With Higher Fitness

Based on effects of fitness reported in the previous literature (15, 43, 48) and on the positive association between fitness and behavioral outcomes in our study, we performed a subgroup analysis with patients and controls with a high degree of fitness. Demographic and clinical characteristics of this subgroup are depicted in Table 3. Originally, we intended to perform a median split, but three patients were in the median range of VO_2peak_ values. In order to achieve a higher statistical power, we decided to assign them to the higher fit subgroup. Consequently, we used the upper 60% (*n* = 12 per group) of the original sample which corresponds to a VO_2peak_ % ranking equal or higher than 35 for the patients and equal or higher than 50 for controls. However, we performed the same analyses excluding those three patients (*n* = 9 per group) and obtained similar results.

**Table 3:** Demographic and clinical characteristics among subgroups with higher degrees of cardiorespiratory fitness.

**Variable**	**ADHD (*n* = 12) mean ± SD**	**Controls (*n* = 12) mean ± SD**	**Statistic**	***P*-value**
Age (years)	31.8 ± 9.2	29.0 ± 7.7	*t* = 0.80	0.44
Gender (f/m)	3/9	2/10	X^2^ = 0.25	0.61
BMI (kg/m^2^)	23.5 ± 2.8	23.5 ± 2.2	*t* = 0.016	0.99
HR_max_ (beats/min)	177.9 ± 11.0	186.9 ± 11.7	*t* = −1.94	0.065
VO_2peak_ (mL/min/kg)	41.0 ± 6.1	46.3 ± 4.4	*t* = −2.46	0.022^*^
VO_2peak_ (% ranking)	55.0 ± 16.0	65.4 ± 11.8	*t* = −1.82	0.082
BDI	9.0 ± 6.6	1.6 ± 1.9	*t* = 3.73	0.001^*^
ADHS-SB	28.4 ± 8.8	5.3 ± 5.0	*t* = 7.92	<0.001^*^
WURS-k	34.8 ± 16.0	9.2 ± 6.8	*t* = 5.11	<0.001^*^
PA total score	7,825 ± 7,375	5,385 ± 3,906	*t* = 1.01	0.33
PA work	3,400 ± 6,204	311 ± 817	*t* = 1.71	0.11
PA transportation	1,956 ± 3,083	1226 ± 968	*t* = 0.78	0.44
PA domestic	691 ± 706	1525 ± 3059	*t* = –0.92	0.37
PA leisure	1,778 ± 1,923	2,323 ± 2,189	*t* = –0.65	0.52
PA walking	1,684 ± 1,944	718 ± 907	*t* = 1.56	0.13
PA moderate	3,360 ± 3,621	3,010 ± 2,927	*t* = 0.26	0.80
PA vigorous	2,780 ± 5,341	1,657 ± 2,258	*t* = 0.67	0.51
Stimulant medication^a^	*n* = 1	—	—	—

* *p <0.05*.

a *Current medication, discontinued 48 h prior to each visit. BMI, body mass index; HR_max_, maximal heart rate as assessed by maximal exercise test; VO_2peak_, peak oxygen consumption as tested by maximal exercise test; VO_2peak_ (% ranking), peak oxygen uptake transformed into age- and gender-adapted percentiles; BDI = Beck Depression Inventory; ADHS-SB, ADHD Self Rating Scale; WURS-k, Wender Utah Rating Scale, retrospective assessment of childhood ADHD; PA, Physical activity as assessed by the International Physical Activity Questionnaire, expressed in MET-minutes/week*.

#### Behavioral Results

Behavioral results for the higher fit sample were similar to the results presented for the complete sample. We found interaction effects between group and condition for reaction times in congruent and incongruent trials [congruent: *F*_(1, 22)_ = 14.78, *p* = 0.001, η^2^ = 0.40; incongruent: *F*_(1, 22)_ = 7.47, *p* = 0.012, η^2^ = 0.25], which were due to enhanced performance in patients in the exercise compared to the control condition. The interaction for the interference score was no longer significant after Bonferroni correction [*F*_(1, 22)_ = 4.74, *p* = 0.040, η^2^ = 0.18]. In explorative analyses, we found interaction effects between group and condition for reaction time variability [congruent: *F*_(1, 22)_ = 17.46, *p* < 0.001, η^2^ = 0.44; incongruent: *F*_(1, 22)_ = 4.67, *p* = 0.042, η^2^ = 0.18] and omissions [*F*_(1, 22)_ = 7.78, *p* = 0.011, η^2^ = 0.27], which were again caused by enhanced performance in patients in the exercise compared to the control condition.

#### fMRI Results

For the flanker task, results including only participants with a high fitness level differed from results for the complete sample. For the contrast incongruent–congruent, there were no interaction effects between group and condition or within-group effects of condition. However, for the single contrasts congruent and incongruent, the subgroup analyses revealed significant within-group effects for the factor condition for ADHD patients but not for controls (Table 4).

**Table 4:** Brain activation differences during the flanker task for Movie—Exercise in higher fit patients with ADHD (*n* = 12).

**Contrast**	**Region of peak activation**	**MNI coordinates (x, y, z)**	**Cluster size**	**t-statistic**	**z-statistic**	p^*^
Congruent	R precentral	30, −4, 46	332	9.80	4.91	<0.001
	R middle temporal	50, −66, 6	181	6.90	4.21	0.007
Incongruent	Frontal superior medial	2, 40, 46	163	7.34	4.33	0.009
	R middle frontal	38, −4, 52	225	6.69	4.14	0.001
	Paracentral lobule	−4, −20, 74	139	5.29	3.66	0.019

* *FWE-corrected on cluster level (initial voxel threshold 0.001 uncorrected)*.

**Figure 3:**
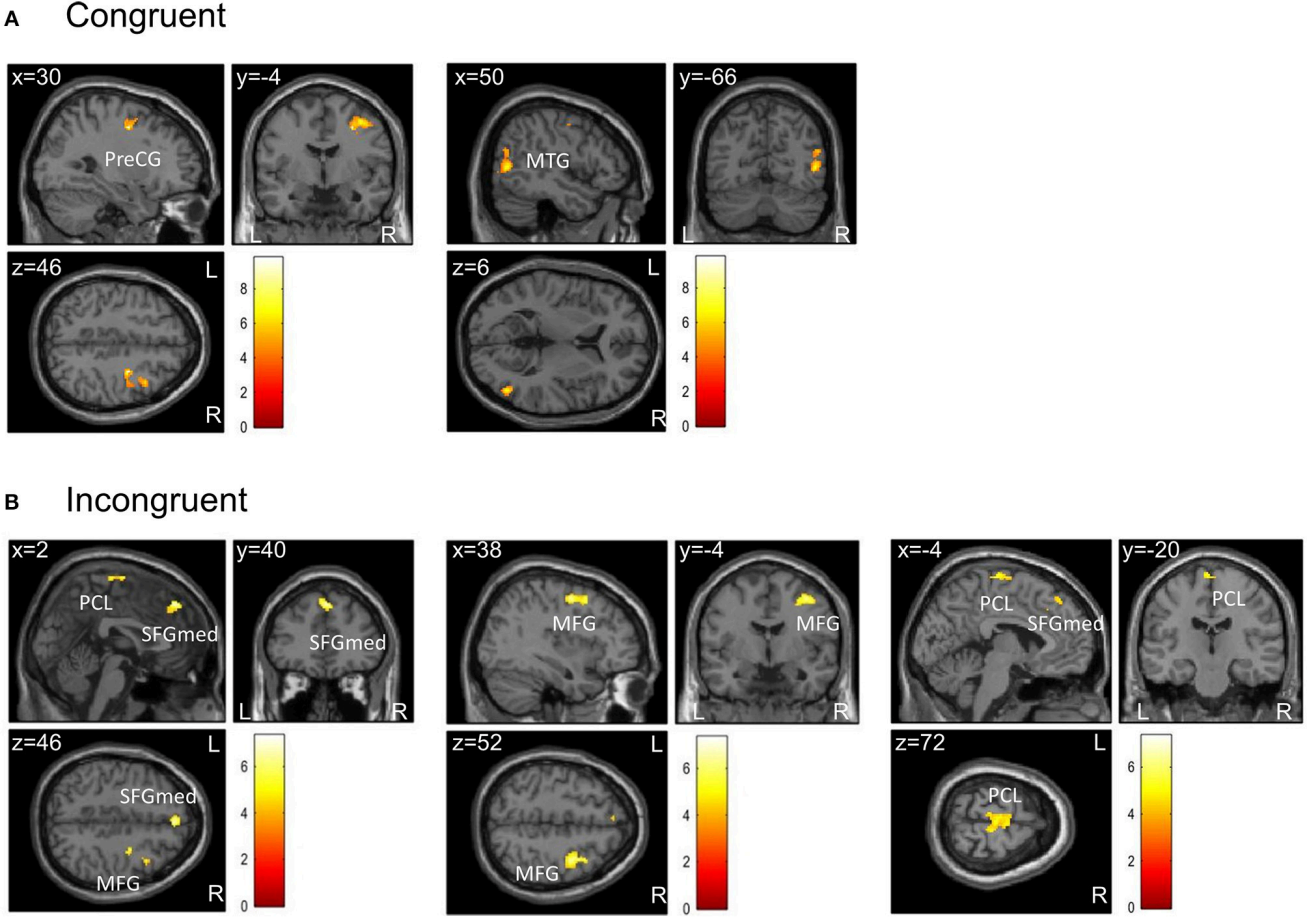
Clusters showing differences in brain activation during **(A)** congruent and **(B)** incongruent trials of the flanker task between the two conditions in higher fit patients (*n* = 12) at the level *p* < 0.05 (FWE-corrected on cluster level, initial voxel threshold 0.001 uncorrected). Colored areas indicate greater activation in the control condition (Movie) than in the exercise condition. **(A)** In congruent trials, activation differences were found in two clusters with peak activation in the right precentral gyrus (PreCG) and right middle temporal gyrus (MTG). **(B)** In incongruent trials, activation differences were found in three clusters with peak activation in the medial part of the superior frontal gyrus (SFGmed), right middle frontal gyrus (MFG), and the paracentral lobule (PCL).

During congruent trials, ADHD patients showed decreased activation in the exercise compared to the control condition in two clusters: one cluster was located in the right precentral gyrus extending to the right superior frontal gyrus and the right middle frontal gyrus (lateral part of premotor cortex); the other cluster comprised regions of the right middle temporal gyrus, right middle occipital gyrus, and right inferior occipital gyrus (Figure 3A).

During incongruent trials, patients showed decreased activation in the exercise compared to the control condition in three clusters: the first cluster extended from the medial to the left lateral part of the superior frontal gyrus, the right midcingulate cortex, and right dorsolateral prefrontal areas; the second cluster extended from the right middle frontal gyrus to right precentral and superior frontal regions (lateral part of premotor cortex); the third cluster was located in the paracentral lobule including the supplementary motor area (medial part of premotor cortex) (Figure 3B).

### Visual Task

**Figure 4:**
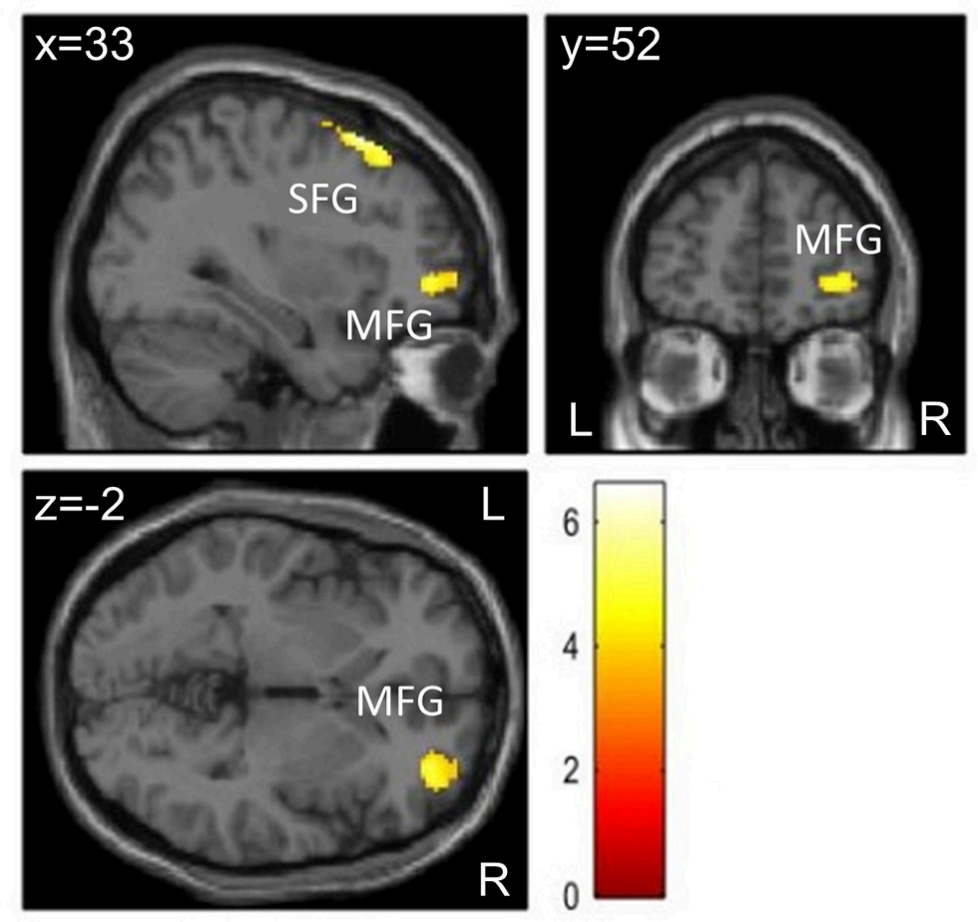
Brain activation differences during visual stimulation at T1 for Movie – Exercise in patients with ADHD (*n* = 20) at the level *p* < 0.05 (FWE-corrected on cluster level, initial voxel threshold 0.001 uncorrected). Activation differences were found in two clusters with peak activation in the right superior frontal gyrus (SFG) and right middle frontal gyrus (MFG).

For the visual task presented directly after each condition (T1), there were no interaction effects between group and condition. However, patients showed decreased activation in the exercise compared to the control condition in two clusters in the right hemisphere: one cluster had peak activation in the superior frontal gyrus and comprised the supplementary motor area and frontal eye fields; the second cluster was located in the anterior/frontopolar part of the prefrontal cortex and comprised parts of the middle and superior frontal gyri (Figure 4 and Table 5). There was no change in activation for healthy controls associated with exercise. We found no effect of condition for the visual task at T2 as well as no interaction effects between condition (Movie and Exercise) and time of visual task (T1 and T2).

**Table 5:** Brain activation differences during visual stimulation at T1 for Movie—Exercise in patients with ADHD (*n* = 20).

**Region of peak activation**	**MNI coordinates (x, y, z)**	**Cluster Size**	***t*-statistic**	***z*-statistic**	p^*^
R superior frontal	26, 22, 60	408	6.59	4.70	<0.001
R middle frontal	34, 50,−2	187	5.39	4.15	0.026

* *FWE-corrected on cluster level (initial voxel threshold 0.001 uncorrected)*.

## Discussion

The current study aimed to investigate the effects of acute aerobic exercise on executive function in adult patients with ADHD and healthy controls, using a flanker task. Our main finding was that the exercise compared to the control condition significantly improved reaction times in patients with ADHD but not in healthy controls. Contrary to our expectations, changes in brain activation due to exercise were not obtained for the total sample of participants. However, analyzing subgroups of patients and healthy controls with a higher degree of fitness, we found changes in brain activation in the exercise compared to the control condition only for ADHD patients. For this subgroup, decreased activation was found in frontal brain regions, both for congruent and incongruent trials of the flanker task. Moreover, these patients revealed the same behavioral improvements as shown for the total ADHD sample, whereas no behavioral improvements could be shown for the healthy control group.

### Behavioral Results

Patients with ADHD improved in reaction times in congruent and incongruent trials of the flanker task after exercise, indicating improved attention and processing speed. Interference scores, which are typically used to measure interference control in flanker task performance, were not different between the two conditions, due to improved reaction times in both, congruent and incongruent trials. Exploratory analyses revealed that the amount of omissions and errors did not change after exercise, indicating that the changes in reaction times were not due to a change in speed-accuracy trade-off. In addition, reaction time variabilities, which are typically increased in ADHD (66), improved due to exercise, likewise suggesting improved attention and stability in reaction time performance.

Our study is the first to demonstrate, in a well-controlled design (i.e., counterbalanced and including a healthy control group), that adults with ADHD benefit from acute exercise. Only two previous studies investigated the behavioral effects of acute exercise in adults with ADHD. Gapin et al. (25) reported exercise-related improvement in Stroop task performance while Fritz and O'Connor (26) did not find changes in vigilance and impulsivity as assessed by the Continuous Performance Task. Both studies had several drawbacks including small sample sizes, paucity of a healthy control group, or pre-exercise post-exercise design which might lead to practice effects. Studies focusing on children with ADHD also revealed mixed results. Two previous studies investigated the effects of acute exercise on interference control using a flanker task. While Pontifex et al. (22) found increased overall accuracy but no change in reaction times after exercise, Ludyga et al. (67) observed decreases in reaction times combined with increases in P3 amplitude as indicators of improved attention. Other studies that assessed Stroop task performance reported enhancements in the Stroop interference condition (21) as well as decreases in reaction times during congruent trials (23), indicating improved interference control and general processing speed, respectively. Our study adds evidence that adults with ADHD can also benefit from exercise.

In contrast to ADHD patients, healthy controls only improved in interference scores after exercise, but note that this effect was due to very small decreases in reaction times for incongruent trials combined with small increases in reaction times in congruent trials. In meta-analytical investigations it has been demonstrated that acute exercise of moderate intensity has overall small to moderate positive effects on many cognitive functions in healthy adults while executive functions, which rely on prefrontal cortex processing, seem to benefit the most (15, 16, 36). However, when focusing on studies of flanker task performance, results range from improvements in terms of reaction times or accuracy (68, 69) to no or even detrimental effects of exercise (70). We assumed that individuals who are impaired in their performance should benefit to a greater extent from exercise. Thus, the lack of an improvement in our healthy control group is contrary to our hypothesis but could be due to ceiling effects as healthy controls responded already faster than patients in the control condition. Previous reviews and meta-analyses demonstrated that children and older adults, i.e., groups with lower cognitive performance levels, are more susceptible to benefits from acute and chronic exercise (15, 30, 51).

### fMRI Results for the Flanker Task

Analyses of brain activation in the single conditions revealed regions similar to those reported to be activated during tasks of interference control in other studies (31, 71, 72). Greater brain activation in incongruent trials than in congruent trials was found in frontal and sensorimotor regions for patients and controls, which is highly indicative of an interference effect and the need for executive control in both groups.

#### Exercise-Related Changes in Brain Activation

Contrary to our hypothesis, we did not observe exercise-induced changes in brain activation during the flanker task when we examined the whole sample, neither in patients nor in controls. It has to be noted, however, that this is the first study to examine exercise-related changes in brain activation using fMRI in patients with ADHD. Findings of previous studies on healthy controls are mixed. Only two studies implemented a counterbalanced design to test the effects of acute exercise on working memory performance (39, 40). Of those, only one study (39) found improved working memory performance after 35 min of moderate cycling compared to seated rest in preadolescent children, in addition to increased activation in parietal areas, hippocampus, and the cerebellum. However, with nine participants their sample size was really small. Li et al. (40) found effects of 20 min of moderate exercise on brain activation of 15 female college students. Activation in the middle frontal gyrus, the right lingual gyrus, and the left fusiform gyrus increased after exercise compared to seated rest while activation of the anterior cingulate cortex, the left inferior frontal gyrus, and the right paracentral lobule decreased. However, they found no behavioral effects of exercise on working memory performance. MacIntosh et al. (37) tested the effects of exercise on response inhibition using a Go/No-go task in 16 healthy adults and reported decreased activation post exercise compared to pre exercise only in the left parietal operculum, combined with no behavioral changes. The authors did not include a control condition, which leaves the possibility of habituation effects. Rajab et al. (73) conducted the only fMRI study investigating exercise-induced changes in resting-state networks. Interestingly, they observed increased co-activation in auditory, sensorimotor, and thalamic-caudate resting state networks after exercise but no changes in attention and executive networks. In psychiatric patients, the only neuroimaging study on acute effects of exercise on cognition was conducted recently by Metcalfe et al. (41). The authors tested bipolar patients and healthy controls in a task assessing attention and response inhibition before and after 20 min of cycling. Despite a lack of behavioral changes, patients with bipolar disorder compared to healthy controls showed stronger deactivation in frontal and temporal regions, hippocampus, and posterior cingulate cortex during sustained attention post exercise compared to pre exercise. All these studies differed considerably in relevant aspects such as samples, design, and cognitive function assessed, which makes a direct comparison to our study difficult. Furthermore, they did not provide information on participants' fitness level, which might modulate the effects of exercise.

#### Subgroup Analysis of Participants With Higher Fitness

When only focusing on participants with a higher degree of fitness, we observed exercise-induced decreases in patients' brain activation during congruent and incongruent trials of the flanker task in one region comprising the lateral part of the right premotor cortex (BA6), located at the borders between the precentral, superior frontal, and middle frontal gyri. During incongruent trials, two additional clusters in regions of the medial frontal cortex showed decreased activation after exercise. One cluster extended from the medial part of the superior frontal gyrus to the midcingulate cortex and the more lateral part of the superior frontal cortex. The second cluster was located in the paracentral lobule and comprised the supplementary motor area, which constitutes the medial part of premotor cortex. The premotor cortex has previously shown to be involved in executive function tasks, predominantly responsible for preparation, selection, and execution of motor responses, spatial guiding, and learning of rules (74–79). The supplementary motor area in particular has been associated with motor planning and resolution of response conflict (80, 81). The medial frontal cortex is associated with pre-response conflict and decision uncertainty (82). Thus, areas important for motor planning and execution combined with areas associated with cognitive control (i.e., brain regions supporting flanker task performance) showed decreased activation after exercise in patients with ADHD. When interpreting this in light of the behavioral improvements observed, one could argue that exercise may have facilitated motor processes, i.e., processing and response speed, and improved attentional processes. Our results advance previous studies using EEG to examine the influence of cardiorespiratory fitness on acute exercise effects on cognition. Tsai et al. (49, 50) also reported greater benefits from acute moderate exercise as expressed by P3 and CNV components in healthy adults with higher fitness as compared to those with lower fitness. In contrast, Magnié et al. (83) found exercise-related changes in P3 amplitude and latency during an auditory oddball task irrespective of fitness level. It has to be noted, however, that participants exercised at maximal intensity, where fitness can play a different role.

#### Direction of Changes in Brain Activation

Due to mixed results of previous studies investigating exercise effects on brain activation, we did not have a hypothesis concerning the expected direction of changes in brain activation. In addition, studies comparing brain activation during executive function tasks between patients with ADHD and healthy controls reported deviations into both directions. Greater activation in areas indicated in our study (e.g., superior and middle frontal, precentral, supplementary motor area) has been reported for children and adults with ADHD during executive function tasks (13, 14, 84). We could speculate that decreased activation in these areas may implicate “normalization” of deviant activation patterns or a decrease in cognitive effort after exercise in patients with higher fitness, which remains to be investigated in future studies. However, note that in our control condition patients differed from controls only in behavioral performance measures, whereas brain activation differences were not significant. It is also worth to note that all analyses involving cardiorespiratory fitness were explorative and should be interpreted accordingly.

### fMRI Results for the Visual Task

During visual stimulation at T1, only patients showed decreased brain activation in the exercise compared to the control condition in two areas: the right premotor cortex including the supplementary motor area and extending to the frontal eye fields, and the frontopolar part of the right prefrontal cortex. This indicates that exercise influenced visual processing in patients with ADHD. Structural and functional abnormalities in areas related to visual processing in patients with ADHD have been reported before (85–87). Although these were mainly limited to occipital regions, activation of frontal regions during checkerboard stimulation has been observed before in healthy persons, which the authors interpreted as activation related to spatial attention (88). In addition, previous studies using EEG found that different components of the visual evoked potential can be modified by exercise. For instance, exercise reduced latency and modified amplitude of the P1, N145, and P3 components of the visual evoked potential, indicating an effect of exercise on speed of visual processing and on visual attention (89–92). The authors concluded that different stages of stimulus processing, i.e., early perceptual processes as well as late attentional processes can be influenced by exercise (93, 94). Specifically the P1 component has been demonstrated to be modulated depending on how much attention is allocated to the stimulus (94, 95).

Based on these findings, decreased brain activation after exercise in frontal areas could indicate that exercise improved visual attention and sensory processing, and that fewer resources were necessary to allocate attention and to process the stimulus. This interpretation matches the behavioral enhancements during the flanker task which we found in patients. Improvements were mainly present in reaction time measures, i.e., indicators of processing speed and attention. Here, no change in brain activation was found for the complete patient sample, which could implicate that there was no change in the demand for cognitive effort to perform better. As decreased brain activation in frontal areas was found in patients with higher degrees of fitness, those patients might have needed fewer cognitive resources to perform in the flanker task after exercise. In line with this interpretation, we also observed a positive relationship of fitness level and the size of behavioral effects. For the visual task at T2, there was no difference in brain activation between the two conditions, implicating a limited duration of exercise effects.

### Limitations

One limitation was that we were not able to recruit patients from the full spectrum of fitness levels. Only seven patients reached a VO_2peak_ corresponding to a percentile above 50, which represents the mean of the ACSM (64) norm sample. To reach a sample size high enough to analyze, we decided to take the upper 60% as “higher fit” which included patients with VO_2peak_ % ranking values below 50 which in fact might belong to the lower fitness range. Second, our patient sample was quite heterogeneous regarding age and symptom severity and though our sample size was in the range of other patient studies, a greater sample would have been therefore favorable.

## Conclusions

Our study adds evidence that adults with ADHD can benefit from acute exercise. In an experimental context, acute aerobic exercise is of particular relevance because it might cause direct improvements in behavior as well as immediate changes in neurophysiology. As short term effects of acute exercise are likely to accumulate to long term benefits of chronic exercise interventions, our findings are highly relevant for developing further treatment approaches for patients with ADHD. Exercise could help as an adjunctive therapy to existing therapy approaches or in some cases even as a stand-alone therapy. Advantages of exercise include low costs, easy implementation, non-invasiveness, as well as additional psychological and physiological benefits. In addition, the study contributes to clarifying the underlying neurophysiological mechanisms of exercise benefits on cognition. However, determining modifying factors (e.g., time point of testing or exercise duration and intensity) that create optimal conditions to observe improvements due to exercise, will be a challenge of future investigations.

## Data Availability

The raw data supporting the conclusions of this manuscript will be made available by the authors, without undue reservation, to any qualified researcher.

## Ethics Statement

The study was conducted in accordance with the 1964 Helsinki declaration and its later amendments or comparable ethical standards and all procedures were approved by the ethics committee of the University of Oldenburg. Informed consent: Informed consent was obtained from all individual participants included in the study.

## Author Contributions

AM designed the study, carried out and supervised data collection, carried out final data analysis, drafted the initial manuscript, and approved the final manuscript as submitted. JÖ designed the study, participated in final data analysis, critically reviewed and revised the initial manuscript, and approved the final manuscript as submitted. AL coordinated and supervised patient recruitment, carried out clinical data collection, and approved the final manuscript as submitted. MB conceptualized and designed the study, participated in data collection, and approved the final manuscript as submitted. HM gave advice during patient recruitment, data collection and analysis, and approved the final manuscript as submitted. CT designed the study, critically reviewed and revised the initial manuscript, and approved the final manuscript as submitted. AP conceptualized and designed the study, wrote the proposal for intramural funding, supervised clinical data collection and analysis, critically reviewed and revised the initial manuscript, and approved the final manuscript as submitted.

### Conflict of Interest Statement

AL declares that she has received travel grants within the last year from MEDICE Arzneimittel Pütter GmbH and Co. KG; and has authored books and articles on ADHD published by Elsevier, Thieme, Springer, and Oxford Press. AP declares that she served on advisory boards, gave lectures, performed phase 3 studies, or received travel grants within the last 5 years from Eli Lilly and Co, Lundbeck, MEDICE Arzneimittel, Pütter GmbH and Co KG, Novartis, Servier, and Shire; and has authored books and articles on ADHD published by Elsevier, Hogrefe, Schattauer, Kohlhammer, Karger, and Springer. The remaining authors declare that the research was conducted in the absence of any commercial or financial relationships that could be construed as a potential conflict of interest
