# Supplementary material for: Acute Effects of Aerobic Exercise on Executive Function and Attention in Adult Patients With ADHD
Source: Front Psychiatry. 2019 Mar 26;10:132. doi: 10.3389/fpsyt.2019.00132 (PMC6443849; doi:10.3389/fpsyt.2019.00132)
Supplement: Supplementary file 1 [file Data_Sheet_1.pdf]

Supplementary Material

Task-related brain activation within the single conditions

Supplementary Figure 1 shows condition-specific brain activation related to interference control (contrast incongruent–congruent) for patients and healthy controls.

In the control condition (Movie), patients showed brain activation in three clusters in the left hemisphere: the first cluster extended from the superior parietal cortex to inferior parietal cortex and postcentral gyrus; the second had peak activation in the precentral gyrus and extended to superior and middle frontal gyri, supplementary motor area, paracentral lobule, and postcentral gyrus; the third comprised postcentral, inferior parietal, and supramarginal regions.

In the exercise condition, activations for patients were observed in two clusters: one cluster was located in the right precentral gyrus, extending to superior and middle frontal gyri and the supplementary motor area; the other cluster comprised left precentral, postcentral, and superior frontal regions.

In the control condition, healthy controls showed brain activation for the contrast incongruent – congruent in six clusters: the first cluster had peak activation in the (left) supplementary motor area and extended to frontal areas of both hemispheres (anterior and mid cingulate, superior and middle frontal, precentral) as well as parietal regions of the left hemisphere (superior and inferior parietal, postcentral, paracentral, precuneus); the second cluster extended from the right precuneus to right occipital and parietal regions; the third and fourth cluster comprised occipital and temporal areas of the right and left hemisphere respectively; the fifth cluster was located in the left insula, extending to left superior temporal pole, rolandic operculum, and inferior frontal operculum; the last cluster comprised the left precentral gyrus and the inferior frontal operculum.

In the exercise condition, healthy controls showed activation in three clusters: the first cluster extended from the left postcentral gyrus to left superior and inferior parietal cortex as well as left precentral gyrus; the second cluster comprised left superior and middle frontal and precentral regions; the third cluster had peak activation in the right middle frontal gyrus, extending to right superior frontal and precentral gyri.

There were no significant group differences in brain activation in the control or exercise condition.

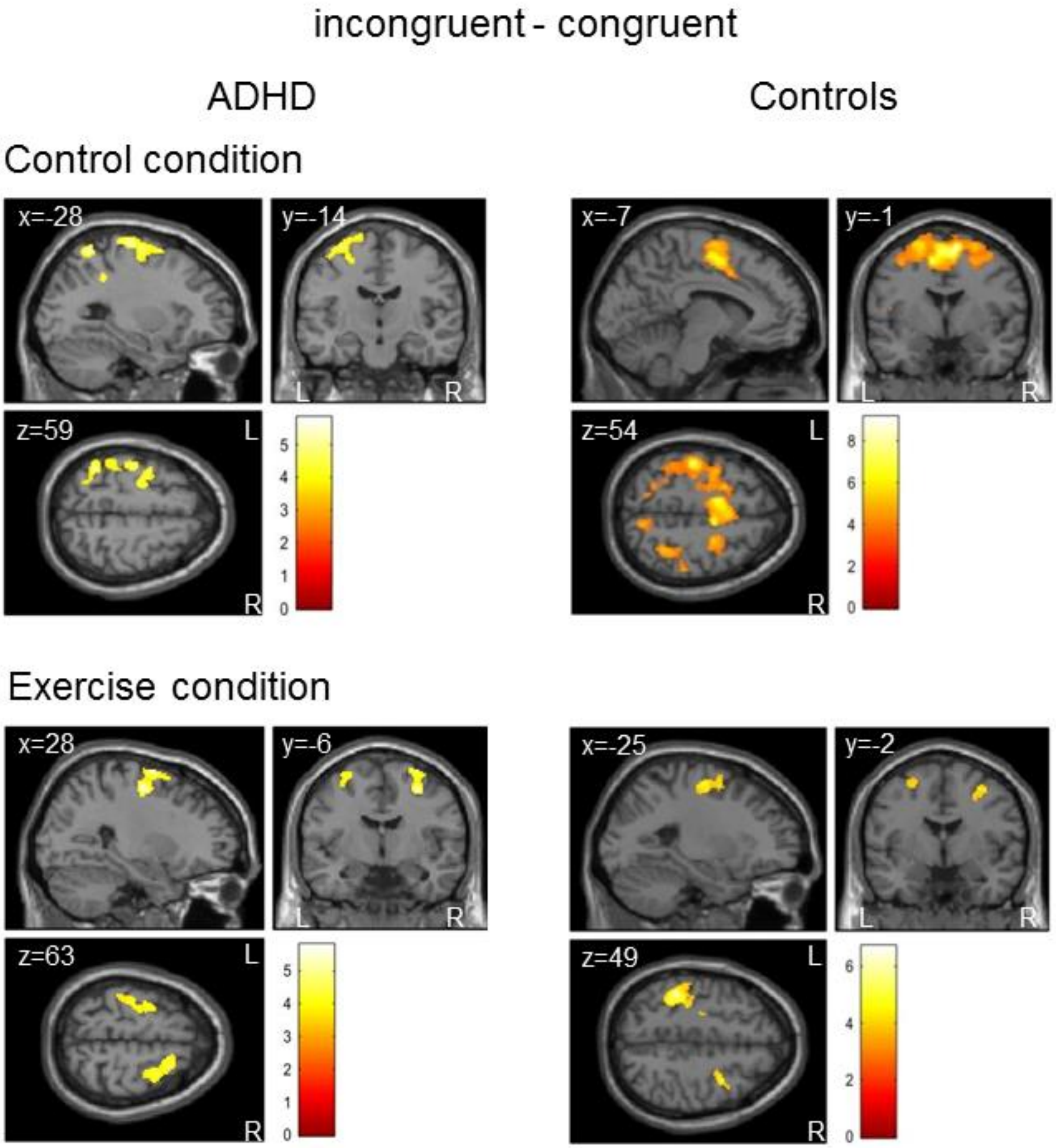

Supplementary Figure 1. Brain activation during the flanker task for the contrast incongruent – congruent in the exercise condition and the control condition for patients and healthy controls at the level $p<0.05$ (FWE-corrected on cluster level, initial voxel threshold 0.001 uncorrected).
